# Tendon‐to‐bone healing after repairing full‐thickness rotator cuff tear with a triple‐loaded single‐row method in young patients

**DOI:** 10.1186/s12891-021-04184-x

**Published:** 2021-03-26

**Authors:** He-Bei He, Tao Wang, Min-Cong Wang, Hui-Feng Zhu, Yue Meng, Cheng-Long Pan, Yong Hu, Xiao-Min Chao, Chun Yang Yang, Min Wang, Jian Feng Ou-Yang

**Affiliations:** 1 Department of Joint Surgery, the Fifth Affiliated Hospital of Southern Medical University, Guangdong Province Guangzhou, China; 2 Department of Orthopedics, The Third Affiliated Hospital of GuangZhou Medical University, Guangdong Province Guangzhou, China; 3 Department of Orthopedics, Guangzhou Red Cross Hospital, Guangdong Province Guangzhou, China; 4 Department of Orthopedics, Zhuhai People’s hospital, Guangdong Province Zhuhai City, China

**Keywords:** Rotator cuff tear, Arthroscopic repair, Single‐row, Regeneration, Bone marrow vents

## Abstract

**Background:**

Arthroscopic repair is recommended for young patients with full-thickness rotator cuff tears (RCTs), but the healing rates have raised concerns. The Southern California Orthopedic Institute (SCOI) row method has been developed based on greater than 3 decades of experience with excellent clinical outcomes; however, studies with a focus on the younger patient population are limited in number. The current study assessed the short-term clinical outcome and the initial tendon-to-bone healing in a young cohort after repair of a full-thickness RCT using the SCOI row method.

**Methods:**

A retrospective cohort study was performed. Patients < 55 years of age who had a full-thickness RCT and underwent an arthroscopic repair using the SCOI row method were reviewed. Clinical outcomes were assessed at baseline, and 3 and 6 months post-operatively. The visual analog scale (VAS), University of California at Los Angeles (UCLA) scale, and Constant-Murley score were completed to assess pain and function. Active range of motion was also examined, including abduction and flexion of the involved shoulder. A preoperative MRI was obtained to assess the condition of the torn tendon, while 3- and 6-month postoperative MRIs were obtained to assess tendon-to-bone healing. Repeated measurement ANOVA and chi-square tests were used as indicated.

**Results:**

Eighty-nine patients (57 males and 32 females) with a mean age of 44.1 ± 8.6 years who met the criteria were included in the study. Compared with baseline, clinical outcomes were significantly improved 3 and 6 months postoperatively based on improvement in the VAS, UCLA score, and Constant-Murley score, as well as range of motion. Greater improvement was also noted at the 6-month postoperative assessment compared to the 3-month postoperative assessment. Three- and six-month postoperative MRIs demonstrated intact repairs in all shoulders and footprint regeneration, which supported satisfactory tendon-to-bone healing. The mean thickness of regeneration tissue was 7.35 ± 0.76 and 7.75 ± 0.79 mm as measured from the 3- and 6-month MRI (P = 0.002). The total satisfactory rate was 93.3 %.

**Conclusions:**

Arthroscopic primary rotator cuff repair of a full-thickness RCT using the SCOI row method in patients < 55 years of age yields favorable clinical outcomes and early footprint regeneration.

## Introduction

A rotator cuff tear (RCT) is a common disorder associated with pain and dysfunction in the shoulder, the prevalence of which increases with age [1]. Full-thickness RCTs are present in approximately 25 % of individuals in their 60 s and 50 % of individuals in their 80 s; however, the reported incidence is lower for patients < 55 years of age (4-8 %) [1, 2]. RCTs in older patients are predominantly attributed to degenerative tears. Younger patients can present with degenerative tears, but the tears commonly result from traumatic injuries and lesions secondary to calcium deposits [3]. The supraspinatus tendon is involved most often because the supraspinatus tendon bears the majority of shoulder-stabilizing strains and has unique anatomic constraints that render it more susceptible to injury [4]. Therefore, features, such as the tear size, pattern, and disease duration, are variable. Younger patients tend to have higher expectations for proper joint function than older patients because of greater family responsibilities [5]. Thus, younger patients always hope for an early recovery and return to work. Moreover, younger patients have several biologic and mechanical factors that favor a successful rotator cuff repair, such as tendon quality and vascularity [6]. With these potentially significant differences between older and younger rotator cuff patients, studies focusing on young patients are warranted; however, such studies are limited in number.

Tendon-to-bone healing contributes to increased strength, increased function, and higher patient- and physician-derived outcome scores [7]. Many factors are associated with rotator cuff tendon healing following repair, including increased age, tear size, tendon and bone degenerative changes, and gap formation between the repaired tendon and the bony insertion shortly after surgical repair [8–10]. Although advances in surgical skills, techniques, technology, and equipment have significantly facilitated the arthroscopic repair of full-thickness RCTs, failed repairs are frequently reported [11–14]. Indeed, approximately 40 % of patients with RCTs have anatomic failure after an arthroscopic repair [11–14]. Such a failure rate is clearly not acceptable for young patients. Snyder [15] proposed that biologic repair of all living tissues consistently requires 5 key elements: stabilization; inflammation; revascularization; cellular repopulation; and remodeling. Thus, the Southern California Orthopedic Institute (SCOI) row has been developed based on over 3 decades of experience with a reported > 90 % healing rate [16].

The SCOI row method utilizes a medially-based single-row anchor with 3 high-strength sutures to securely fix the cuff with minimal tension, and small puncture holes or bone marrow vents in the prepared tuberosity that facilitate flow of the bone marrow, thus covering the repaired cuff and forming a healing blanket. Advocates of the SCOI row method stress the evidence of tendon-to-bone healing, which is supported by the large footprint regeneration area and complete restoration of the rotator cuff after repair [15]. Theoretically, the younger population has superior biologic and mechanical advantages that facilitate healing after repair [6]. Thus, the SCOI row method might be the optimal treatment option for young patients with a full-thickness RCT. A retrospective study by Burns and Synder [17] enrolled young patients (< 50 years of age) who underwent arthroscopic repair using the SCOI row method with a minimum 3-year follow-up and reported satisfactory clinical outcomes; however, images demonstrating tendon-to-bone healing were lacking in the study. Young patients always hope for early recovery and return to work postoperatively, thus the short-term clinical outcomes and early tendon-to-bone healing were our concerns. The current study determined the clinical outcomes and tendon-to-bone healing rates based on MRIs in young patients with medium-to-large full-thickness RCTs who underwent repair using the SCOI row method.

## Methods

### Patients

This was a retrospective study. The study was approved by the local Institutional Review Board (2019-GJWK-01). All procedures involving human participants were in accordance with the ethical standards of the Institutional and/or National Research Committee and with the 1964 Helsinki Declaration and its later amendments or comparable ethical standards.

The medical records of all patients who underwent an arthroscopic repair of a full-thickness RCT before March 2020 were reviewed. The inclusion criteria were as follows: (1) symptomatic full-thickness RCT based on MRI findings and refractory to conservative treatment, including 6 weeks of physical therapy, non-steroidal anti-inflammatories, and activity modification; (2) tear size between 2 and 5 cm in the anterior-to-posterior dimension measured during arthroscopy; (3) only the supraspinatus tendon was sutured and repaired; (4) primary repair using the SCOI row method with a single‑row technique augmented by bone marrow vents; (5) a minimum of 6 months of follow-up evaluations; (6) the operation performed by the same surgeon; and (7) < 55 years of age at the time of surgery.

The exclusion criteria were as follows: (1) RCTs that were deemed irreparable during surgery, based on the predictors reported by Kim et al. (chronic pseudoparalysis, acromiohumeral distance, large mediolateral tear size, positive tangent sign, high-grade fatty degeneration of the supraspinatus, and three or four tendon tears) [18]; (2) RCTs requiring interval slides and/or margin convergence sutures; (3) RCTs requiring anchor fixation of the subscapularis tendon; and (4) concomitant injuries in the involved shoulder, such as a Bankart or Hill-Sachs lesion.

### Surgical Procedure

**Fig. 1:**
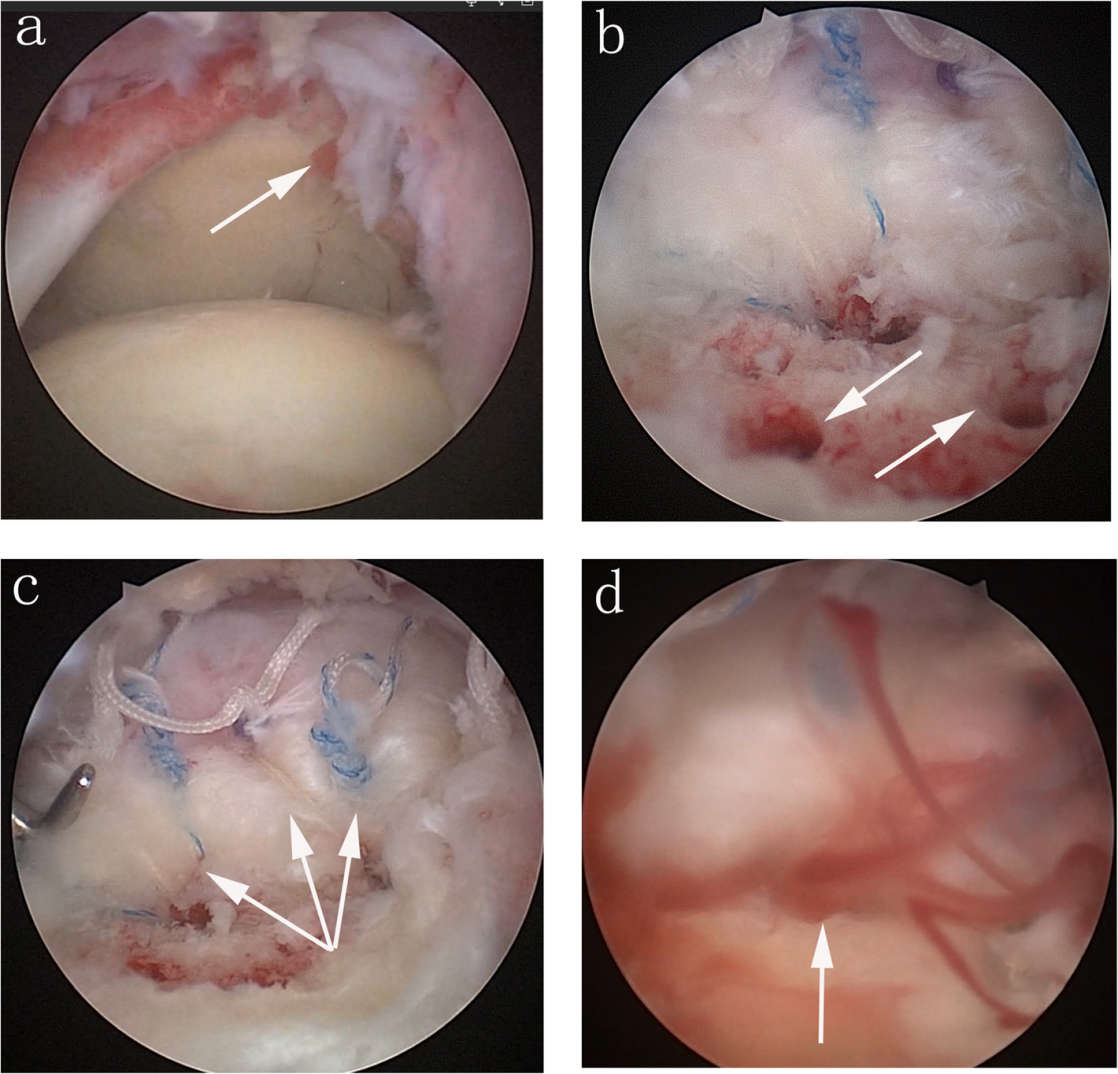
Arthroscopic view of the rotator cuff repair. Picture **a** shows the large size full-thickness torn supraspinatus tendon with a U-shape. Picture **b** shows the bone morrow vents in the greater tuberosity after debridement of the remaining tendon. Picture **c** shows that the sutures passed the tendon in a “fan-like” array and were tied with locking sliding knots. Picture **d** shows the emerging crimson duvet bone marrow enveloping the footprint

All procedures were performed under general anesthesia. The patient was placed in the lateral decubitus position with the arm in 70 degrees of abduction and 15 degrees of forward flexion. Posterior and anterior mid-glenoid portals were created to enter the glenohumeral compartment and probe the pathology of the biceps and subscapular tendons. An arthroscopic biceps tenotomy and tenodesis were performed if biceps tenosynovitis was involved. A mid-lateral subacromial portal was created to enter the subacromial space. Subacromial decompression was performed if signs of coracoacromial ligament undersurface mechanical abrasion were present. An acromioplasty was performed with subacromial decompression if an impingement syndrome was significant. The tear pattern and size were arthroscopically-assessed via the lateral portal based on previous descriptions [19, 20] (Fig. 1a).

**Fig. 2:**
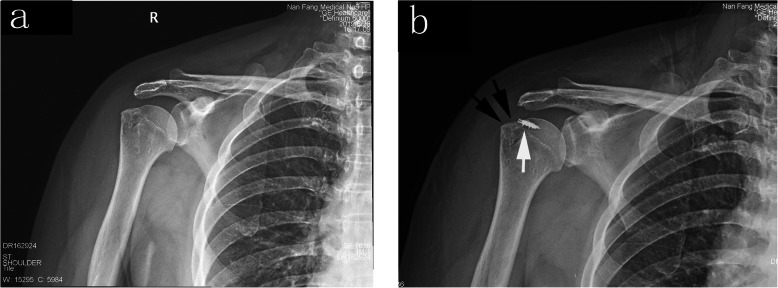
Anterior view of the radiograph. The anchor was inserted 5 mm lateral to the articular cartilage at a 45 degree angle (white arrow). Seven to nine bone marrow vents were created in the greater tuberosity (black arrow)

During the repair, the arm was placed in 45 degrees of abduction and neutral rotation. Debridement and trimming of the infraspinatus tendon were at the discretion of the surgeon if the tendon was partially torn. The torn supraspinatus tendon was debrided to a stable edge using a motorized shaver and anatomic footprint soft tissues were debrided to bare bone. One or two anchors (Corkscrew® FT Anchor, Arthrex, Florida, USA) with three high-strength sutures were used based upon the tear size and pattern. Anchors were inserted into the prepared bone 5 mm lateral to the articular cartilage at a 45-degree angle to the subchondral bone [16] (Fig. 2). This specific angle is recommended to achieve the strongest fixation of the cuff edge and best resistance to anchor pull-out [21]. The anchor was seated with the eyelet facing the cuff tendon. A microfracture awl (Microrevo Punch, Linvatec, Florida, USA) was used to create bone marrow vents with fat globules emerging. Seven-to-nine vent holes were made in the tuberosity beginning several millimeters away from the anchor pilot holes, which were approximately 1.5 cm deep and aimed down the humeral shaft [16] (Fig. 1b). The rotator cuff repair was performed utilizing a standard shuttle technique with the sutures passed as simple stitches in a “fan-like” array [22]. All sutures were tied with locking sliding knots placed over the cuff and followed by three reverse half-hitches on alternating posts [22] (Fig. 1c). After tying all the knots, the arthroscope concentrated on viewing the final repair and assessing the tension. The emerging crimson duvet bone marrow enveloping the footprint was observed when the fluid inflow was suspended (Fig. 1d).

### Postoperative rehabilitation

**S**tandard rehabilitation instructions were supervised by a physical therapist, as suggested by Dierckman et al.[23] The involved arm was immobilized in a neutral rotation sling for 4–5 weeks. On postoperative day 1, active elbow, wrist, and hand exercises, as well as shoulder shrugs, were allowed. During the 1st-6th weeks postoperatively, passive supine external rotation, pendulum exercises, and abduction and forward flexion exercises were carried out, followed by a gradual increase in active motion. Strengthening was initiated at 8 weeks. Generally, patients were permitted to resume full, unrestricted activities 16–20 weeks postoperatively if an intact repair and primary tendon-to-bone healing were demonstrated shown on the 3-month postoperative MRI. Heavy lifting and returning to sports activities were delayed until 6 months based on MRI findings.

### Patient evaluation

Each patient was assessed at baseline, and 3 and 6 months post-operatively. All participants enrolled in the study completed the follow-up evaluations. The visual analog scale (VAS), University of California at Los Angeles (UCLA) scale, and Constant-Murley score were completed to assess pain and function. Active range of motion, including abduction and flexion of the involved shoulder, was also examined preoperatively and at each follow-up evaluation. Preoperative magnetic resonance imaging (MRI) was performed to confirm an intact tendon repair, while 3- and 6-month postoperative MRIs were obtained to assess tendon-to-bone healing. The regeneration footprint thickness was measured as the distance from the anchor insertion site lateral to the regeneration tissue on MRI. The tear pattern and size were confirmed by arthroscopy [19, 20]. At 6 months postoperatively, patients were asked to complete a questionnaire (global assessment) that queries remission/function and satisfaction pre- and post-operatively to determine the efficacy of the SCOI row[24].

### Statistical analysis

All statistical analyses were performed using SPSS for Windows (version 23.0). Continuous data are presented as the mean ± standard deviation (SD). Repeated measurement ANOVA and chi-square tests were used, as indicated. A *P* < 0.05 was considered statistically significant.

## Results

### Patient demographics

Eighty-nine patients who met the criteria were included in the study, including 57 males (64.0 %) and 32 females (36.0 %). The mean age was 44.1 ± 8.6 years, with a range from 23 to 55 years. The longest and shortest disease duration was 17 and 6 weeks, respectively, with a mean disease duration of 10.6 ± 3.4 weeks. Fifty patients (56.1 %) had a history of tobacco use history; 39 patients denied tobacco use. Patients were discharged from the hospital according to standard criteria, as follows: no fever; pain controlled by level 1 or 2 analgesics; an understanding of and taking responsibility for re-tear risks; and familiarity with the rehabilitation protocol. The average in-hospital length of stay postoperatively was 2.2 ± 1.7 days.

Of the patients, 96.6 % (86 of 89) claimed a traumatic etiology. Specifically, 60.5 % (52 of 86) were sustained while lifting heavy objects, 11.6 % (10 of 86) were involved in a traffic accident, 12.8 % (11 of 86) resulted from athletic events, and 15.1 % (13 of 86) were attributed to a fall. Based on the MRI and arthroscopy findings, 67 patients (75.3 %) had a supraspinatus tendon tear alone and 22 patients (24.7 %) had a concomitant injury involving the infraspinatus tendon. The tear size was medium (2–3 cm) in 37 patients and large (3–5 cm) in 52 patients [20]. There were 50 and 39 crescent- and U-shaped tears of the supraspinatus tendon, respectively [19].

### Concomitant arthroscopic procedures

Additional surgical procedures was determined based on the preoperative clinical and intraoperative diagnostic examination findings. Fifty-two of 89 patients (58.4 %) needed subacromial decompression, of whom 37 underwent acromioplasty when an anterior spur was present or additional space was required for visualization or instrumentation in the subacromial space. Biceps tenotomy and tenodesis were performed on 10 patients (11.2 %) with anterior shoulder pain, pain with palpation over the bicipital groove, and MRI and arthroscopic evidence. Twenty patients (22.5 %) underwent debridement of partial-thickness infraspinatus tears according to the tear thickness.

### Complications

There were no infections, neurovascular injuries, revision procedures for shoulder stiffness, or other complications requiring repeat surgical intervention.

### Clinical outcomes

Compared to baseline, clinical outcomes were significantly improved at the 3-month postoperative assessment based on remission in the VAS (mean, -5.621; 95 % CI, -6.102 to -5.139; *P* < 0.001), and increase in the UCLA score (mean, 19.483; 95 % CI, 18.113 to 20.853; *P* < 0.001) and Constant-Murley score (mean, 38.276; 95 % CI, 32.877 to 43.674; *P* < 0.001). Patients also had a significant improvement in the VAS (mean, -6.552; 95 % CI, -7.024 to -6.079; *P* < 0.001), UCLA score (mean, 20.759; 95 % CI, 19.478 to 22.039; *P* < 0.001), and Constant-Murley score (mean, 43.552; 95 % CI, 37.989 to 49.115; *P* < 0.001) at the 6-month follow-up evaluation compared to baseline. Active range of motion, including abduction (mean, 82.931; 95 % CI, 69.146 to 96.716; *P* < 0.001) and forward flexion (mean, 77.241; 95 % CI, 65.801 to 88.682; *P* < 0.001), 3 months postoperatively were much improved compared to baseline. Similar results were demonstrated between baseline and the 6-month follow-up evaluation, with mean differences of 90.172 (95 % CI, 76.036 to 104.309; *P* < 0.001) in abduction and 84.897 (95 % CI, 74.352 to 95.442; *P* < 0.001) in forward flexion.

The VAS (mean, -0.931; 95 % CI -1.337 to -0.525; *P* < 0.001), UCLA score (mean, 1.276; 95 % CI, 0.317 to 2.234; *P* = 0.011), and Constant-Murley score (mean, 5.276; 95 % CI, 2.439 to 8.113; *P* = 0.011) were more improved at 6 months compared to 3 months postoperatively. A significant difference between the 3- and 6-month postoperative assessment was also noted in active range of motion with respect to abduction (mean, 7.241; 95 % CI, 1.151 to 13.332; *P* = 0.022) and forward flexion (mean, 7.655; 95 % CI 1.911 to 13.399; *P* = 0.011); the results are shown in Table 1.

**Table 1:** Clinical outcome comparison

	Baseline	3-month follow-up	6-month follow-up	*P1*	*P2*	*P3*
VAS	6.83 ± 1.167	1.21 ± 0.861	0.28 ± 0.455	< 0.001	< 0.001	< 0.001
UCLA score	12.72 ± 3.250	32.21 ± 1.859	33.48 ± 1.745	< 0.001	< 0.001	0.011
Constant-Murley score	46.24 ± 11.978	84.52 ± 6.098	89.79 ± 5.978	< 0.001	< 0.001	0.001
Range of active abduction	74.48 ± 32.742	157.41 ± 14.918	164.66 ± 14.936	< 0.001	< 0.001	0.022
Range of active forward flexion	82.24 ± 31.327	159.48 ± 13.386	167.14 ± 12.750	< 0.001	< 0.001	0.011

*P1* comparison between baseline and 3-month follow-up; *P2* comparison between baseline and 6-month follow-up; *P3* comparison between 3-month and 6-month follow-up

**Fig. 3:**
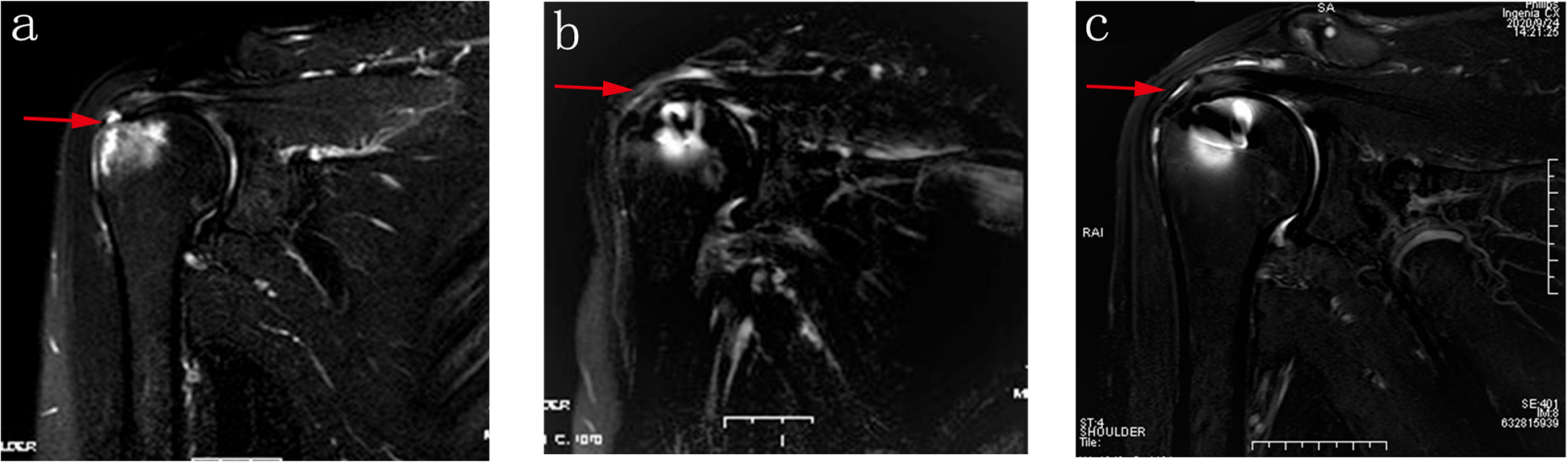
MRI for the rotator cuff. **a** Preoperative imaging revealed the torn supraspinatus tendon (red arrow). **b** Three-month postoperative imaging shows the footprint regeneration (red arrow). **c** Six-month postoperative imaging shows more growth in the footprint (red arrow)

Postoperative MRI demonstrated an intact repair in all shoulders [15]. Furthermore, 3- and 6-month postoperative MRI demonstrated regeneration in the footprint, indicating a good trend in tendon-to-bone healing (Fig. 3). The mean thickness of regeneration tissue was 7.35 ± 0.76 mm as measured on the 3-month postoperative MRI and 7.75 ± 0.79 mm on the 6-month MRI (*P* = 0.002).

At the completion of follow-up, all patients acknowledged pain relief after the repair, of whom 96.6 % had significant pain relief. Most of the patients had improvement in joint function as well. The total satisfactory rate reached 93.3 and 28.1 % of the patients felt very satisfied. The details are shown in Table 2.

**Table 2:** Global assessment at the end of follow-up

	6-month follow-up (*N* = 89)		
	**Values**		**Percentage**
**Pain relief**			
Worse and/or unchanged	0		0 %
Minimally changed	3		10.3 %
Much changed	15		51.7 %
Very much changed	11		38.0 %
**Joint function**			
Worse	0		0 %
Unchanged	3		10.3 %
Improved	26		89.7 %
**Satisfaction**			
Dissatisfied	1		3.4 %
Neutral	3		10.3 %
Satisfied	10		34.5 %
Very satisfied	15		51.2 %

## Discussion

Our results support the findings reported by MacKechnie et al.[5]. Specifically, full-thickness RCTs in patients < 55 years of age respond well to arthroscopic rotator cuff repair, as evidenced postoperatively by good patient-reported outcomes, significant pain relief, improvement in strength, and high satisfaction. Although the duration of follow-up was relatively short in the present study, the UCLA and Constant-Murley scores showed comparable improvement compared to previous studies that involved a younger patient population [6, 17, 25, 26]. In fact, a study with an average follow-up duration of 5.8 years (range, 3.1–13.4 years) conducted by Burns and Synder [17] using the same repair method (SCOI row) suggested an average UCLA score of 32.6 at the end of follow-up period. We report mean UCLA scores 3 and 6 months post-operatively of 32.21 and 33.48, respectively. Comparable results were also achieved with respect to VAS and joint motion range [17]. Therefore, it can be concluded that both the short- and long-term clinical outcomes were favorable for arthroscopic repair using the SCOI row method in young patients with a RCT.

Another important finding of this study was that early tendon-to-bone healing after rotator cuff repair was observed in the MRI for young patients with footprint regeneration in the initial 3 and 6 months postoperatively. The new tissue in the footprint serves as the foundation for strong fixation of the repaired tendon. The reported accuracy of MRI in detection of a healing rotator cuff repair ranges from 86 to 100 % [27, 28]. In the current study, the level of regeneration of the footprint was assessed with MRI. The mean thickness of regeneration tissue was 7.35 ± 0.76 mm when measured in 3-month postoperative MRI, and 7.75 ± 0.79 mm in 6-month MRI, which showed statistical difference (*P* = 0.002). We are of the opinion that the footprint regeneration accounted for the absence of post-operative re-tearing reported by Burns and Synder [17] up to 13.4 years (161 months) postoperatively.

Mechanical advantages and biologic cellular augments of the SCOI row repair have been proposed to facilitate footprint regeneration and improve tendon-to-bone healing rates. Elhassan et al. [27] proposed that the surgical technique, the timing of surgery, tension on the repair, the biomechanical construct, fixation, patches, biologic augments, and the postoperative rehabilitation strategy, affect rotator cuff repair tendon-to-bone healing. Unlike the conventional single-row method, the SCOI row method uses a single row of screw-in suture anchors triple-loaded with high-strength sutures passed as simple stitches in a “fan-like” array [23]. Coons et al. [29] concluded that three simple sutures provide superior suture-tendon security than combinations of one mattress and two simple stitches subjected to cyclic loading.

Although double-row technique has been highlighted for its superior biomechanical properties, such as a larger footprint area, improved initial strength and stiffness, and decreased gap formation and strain [30, 31], Nelson et al. [32] argued that repair strength might be directly related to the total number of sutures passed through the tendon being repaired rather than the number or configuration of suture anchors. Another study conducted by Mazzocca et al. [31] confirmed that the single-row repair technique was similar to the double-row techniques in load-to-failure, cyclic displacement, and gap formation, which was attributed to a larger number of suture passes through the tendon. He et al. [33] reported that rotator cuff repair using the SCOI row method has superior biomechanical properties when compared with the double-row method. Indeed, our results support this view. The current study showed that all rotator cuff tendons had intact repairs at the end of the follow-up period in agreement with the findings reported by Burns and Synder [17]. Kim et al. reported that the repair integrity of RCT treated with arthroscopic single-row with two sutures, double-pulley, and double-mattress suture bridge techniques was 80 %, 87.5 %, 88 % respectively [34]. However, a prospective study with a longer follow-up duration is warranted to determine the long-term healing rate of the SCOI row repair.

In addition to superior biomechanical properties, a medially-based repair with anchors placed near the articular margin of the greater tuberosity has multiple benefits, such as the capability to minimize repaired tendon tension, better screw purchase beneath the subchondral bone, and avoiding lateral shift of the muscle-tendon junction [35]. An *in-vivo* experiment reported by Dierckman et al. [36] suggested that repairing a shortened tendon to the lateral versus medial footprint increases the repair tension 5.4-fold. Animal models have revealed that the decreased modulus of elasticity with increasing tendon tear chronicity might also partially contribute to minimizing the tendon tension [37, 38]. Kim et al. [39] proposed that low-tension repairs promote complete tendon-to-bone healing. On the other hand, the low tension repair reduced the early pain of the involved shoulder after operation, which also helped to decrease analgesic use and length of in hospital. Our patients rarely complained of intolerable pain the first few days postoperatively, which significantly augmented patient satisfaction. The mean in-hospital post-operative length of stay was 2.2 days.

In addition to mechanical advantages, biologic cellular augments contribute to the footprint regeneration. “Bone marrow vents” and the “crimson duvet” from the proximal humeral metaphysis primarily account for footprint regeneration of the rotator cuff despite complete debridement of all soft tissues at the time of repair [15]. This ‘‘super clot crimson duvet’’ is known to contain a rich cache of mesenchymal stem cells, platelets with growth factors and vascular elements, and vascular access channels, all of which will contribute to cuff healing [15, 27]. Nakagawa et al. [40] found that drilling into the footprint improved the quality of repair tissue and biomechanical strength at the tendon-to-bone insertion after rotator cuff repair in an animal model. Milano et al. [41] concluded that tendon-to-bone healing rates were improved from 12.5 % in controls to 60 % with footprint “microfracture” for large cuff tears. Compared to elderly patients, the tendon quality and vascular supply are much better in young patients, which promote tendon healing after repair [42]. These advantages contribute to the rapid footprint regeneration after rotator cuff repair. Thus, the SCOI row method is considered to be the optimal option for young patients with a rotator cuff tear who require surgical repair.

A limitation in this study was the retrospective design. In addition, the small sample size did not allow for robust statistical power. We acknowledge that the short-term follow-up duration (6 months postoperatively) might overestimate the intact repair rate using the SCOI row method. The risk of re-tear might be increased when patients return to the preinjury level of activity and employment. Thus, a study with a longer follow-up duration is warranted to determine the footprint regeneration and intact repair rate. The duration of physical therapy in patients with a rotator cuff tear before surgical repair has not been established. The inclusion criteria (6 weeks of physical therapy) in the current study was shorter than in some studies (3 months).

## Conclusions

Arthroscopic primary repair of full-thickness RCTs with the SCOI row method in patients < 55 years of age provides improved postoperative pain scores, functional outcomes, and satisfaction. The ability to return to work is an important concern in this young patient population, and most of the patients in our study were able to return to their prior level of functioning. Enhanced footprint regeneration was observed in the early period postoperatively, which supports the manifestations of tendon-to-bone healing. Taken together, the SCOI row technique might be the optimal option for young patients with full-thickness medium and large RCTs.

## Data Availability

The datasets used and/or analysed during the current study are available from the corresponding author on reasonable request.

## Abbreviations

RCT: Rotator cuff tear
VAS: Visual analog scale
MRI: Magnetic resonance imaging
SCOI: Southern California Orthopedic Institute
UCLA: University of California at Angeles
